# Impact of small quantity lipid‐based nutrient supplements on infant and young child feeding practices at 18 months of age: results from four randomized controlled trials in Africa

**DOI:** 10.1111/mcn.12377

**Published:** 2016-12-02

**Authors:** Mary Arimond, Souheila Abbeddou, Chiza Kumwenda, Harriet Okronipa, Jaimie Hemsworth, Elizabeth Yakes Jimenez, Eugenia Ocansey, Anna Lartey, Ulla Ashorn, Seth Adu‐Afarwuah, Stephen A. Vosti, Sonja Y. Hess, Kathryn G. Dewey

**Affiliations:** ^1^ Department of Nutrition University of California Davis Davis California USA; ^2^ Department for International Health University of Tampere School of Medicine Tampere Finland; ^3^ Nutrition Group, Department of Epidemiology and Population Health London School of Hygiene and Tropical Medicine London UK; ^4^ Department of Individual, Family, and Community Education University of New Mexico Albuquerque New Mexico USA; ^5^ Department of Family and Community Medicine University of New Mexico Albuquerque New Mexico USA; ^6^ Center for Education Policy Research University of New Mexico Albuquerque New Mexico USA; ^7^ Pacific Institute for Research and Evaluation Albuquerque New Mexico USA; ^8^ Department of Nutrition and Food Science University of Ghana Legon Ghana; ^9^ Department of Agricultural and Resource Economics University of California Davis Davis California USA

**Keywords:** Africa, breastfeeding, feeding practices, infant, nutrient supplements, young child

## Abstract

Optimal infant and young child feeding (IYCF) practices can help ensure nutrient adequacy and support healthy growth and development. Small‐quantity lipid‐based nutrient supplements (SQ‐LNS) have been proposed to help fill nutrient gaps, but little is known about the impact of provision of SQ‐LNS on breastfeeding or complementary feeding practices. In the context of four coordinated randomized controlled nutrient supplementation trials in diverse sites in Africa, we compared IYCF practices at infant age 18 months (after 9–12 months of supplementation) between those receiving and not receiving SQ‐LNS. Practices were assessed by caregiver recall. Continued breastfeeding ranged from 74% (Ghana site) to 97% (Burkina Faso site) and did not differ between groups in any site; prevalence of frequent breastfeeding also did not differ. In two sites (Burkina Faso and Malawi), infants receiving SQ‐LNS were more likely to meet the World Health Organization recommendations for frequency of feeding (percentage point differences of 12–14%, *P* < 0.0001 and *P* = 0.005, respectively; the remaining two sites did not have data for this indicator). Most indicators of infant dietary diversity did not differ between groups in any site, but in the same two sites where frequency of feeding differed, infants receiving SQ‐LNS were less likely to have low frequency of consumption of animal‐source foods in the previous week (percentage point differences of 9–19% for lowest tertile, *P* = .02 and *P* = 0.04, respectively). We conclude that provision of SQ‐LNS did not negatively impact self‐reported IYCF practices and may have positively impacted frequency of feeding.

## 1 INTRODUCTION

The global prevalence of undernutrition among infants and young children is slowly declining, with regional variations. However, recent estimates indicate that 165 million children under 5 years remain stunted and that undernutrition in all forms underlies 3.1 million child deaths annually (Black et al., 2013). Child undernutrition has many causes and no easy or single solution, but suboptimal infant and young child feeding (IYCF) practices constitute a modifiable risk. Recommended practices include early and exclusive breastfeeding to 6 months, continued breastfeeding to 2 years or beyond, and timely introduction of nutritionally adequate and safe complementary food (WHO/UNICEF, 2003). The World Health Organization (WHO) has described indicators for assessing IYCF practices at population or group level (WHO, 2008), covering both breastfeeding and complementary feeding.

Breastfeeding promotion and complementary feeding interventions, including both educational approaches and provision of foods, are identified as core nutrition interventions (Bhutta et al., 2008; Bhutta et al., 2013). One aim of complementary feeding interventions is to ensure that infant diets meet micronutrient needs; a range of micronutrients is often lacking, and complementary foods may also lack sufficient energy density and essential fatty acids (Dewey & Brown, 2003; Michaelsen et al., 2011). Home fortification approaches, including multiple‐micronutrient powders and small‐quantity lipid‐based nutrient supplements (SQ‐LNS), have been proposed as one option for filling nutrient gaps and supporting healthy infant growth and development (de Pee, 2015).

Small‐quantity lipid‐based nutrient supplements can be made from a variety of ingredients; often they are made of peanut, vegetable oil, sugar, and a vitamin/mineral mix, with or without milk powder. They are designed to enrich local foods such as grain‐ or tuber‐based porridges and to fill nutrient gaps during complementary feeding. SQ‐LNS are generally provided in doses of ≤20 g (≤120 kcal) and are intended to not replace or reduce breastfeeding or diversification of infant diets with local foods (Arimond et al., 2015).

Few studies to date have assessed the impact of lipid‐based nutrient supplements (LNS) on IYCF practices, especially after longer periods of supplementation, and concerns have been raised about the potential of LNS to undermine breastfeeding or feeding with local foods (see, e.g., Latham, Jonsson, Sterken, & Kent, 2010; McLellan, 2014). Effects on IYCF practices could be mediated either by maternal perceptions of different needs for breast milk or local foods for infants receiving LNS or by a change in appetite, demand for breastfeeding, or preference for local foods among infants who consume LNS.

This paper describes the impact of provision of LNS on self‐reported IYCF practices in four coordinated, randomized supplementation trials in three African countries, conducted by the International Lipid‐Based Nutrient Supplements (iLiNS) Project research consortium (http://www.iLiNS.org). Infant feeding practices vary across the three countries, with the proportion still breastfed at 20–23 months of age ranging from 50% in Ghana to 77–80% in Malawi and Burkina Faso based on the most recent national surveys (National Statistical Office (NSO) and ICF Macro, 2011; Institut National de la Statistique et de la Démographie et ICF International, 2012; Ghana Statistical Service, Ghana Health Service, and ICF International, 2015). The proportion of infants 6–23 months of age meeting the WHO criteria for frequency of feeding ranged from 39% in Burkina Faso to 43% in Ghana and 54% in Malawi, and only 6% of Burkinabe infants met the WHO criteria for minimum dietary diversity, whereas 28–29% did so in Ghana and Malawi.

The objective of this study was to determine if IYCF practices in our four trials differed between groups who had received or not received SQ‐LNS for 9–12 months, when children were approximately 18 months of age (study endline).

**Key messages**

- The World Health Organization provides guidance on breastfeeding and complementary feeding practices, and suboptimal practices are a modifiable risk for child undernutrition.
- Small‐quantity lipid‐based nutrient supplements are designed to fill nutrient gaps and are designed to not replace or reduce breastfeeding or diversification of infant diets with local foods.
- Provision of small‐quantity lipid‐based nutrient supplements for 9–12 months did not change breastfeeding practices at endline (18 months of age) in four coordinated randomized supplementation trials in Africa.
- Diversification of infant diets was not reduced, while frequency of feeding was positively impacted.

## 2 METHODS

### 2.1 Study designs

Four randomized nutrient supplementation trials were carried out at sites in Burkina Faso, Ghana, and Malawi (two trials). The trials were designed to test the efficacy of provision of SQ‐LNS for promotion of infant growth and development. Trial designs, description of participants, and results for primary outcomes have been previously reported (Adu‐Afarwuah et al., 2015; Ashorn et al., 2015a; Ashorn et al., 2015b; Hess et al., 2015; Maleta et al., 2015) and are briefly described here.

Study designs differed by trial (**Table** 1). The Burkina Faso iLiNS‐ZINC trial (hereafter, “Burkina ZINC”) was a community‐based cluster‐randomized trial designed to identify zinc‐related biochemical and functional responses to 20 g SQ‐LNS containing various amounts of zinc among four intervention groups and also to compare the same outcomes between communities receiving an intervention package including SQ‐LNS and treatment of malaria, fever, and diarrhea and non‐intervention communities (Hess et al., 2015). For the purposes of this analysis, all intervention groups (all receiving 20 g of SQ‐LNS from 9 to 18 months of age) are combined and compared with the non‐intervention group, which did not receive SQ‐LNS until after 18 months of age. All infants were recruited at 9 months of age.

**Table 1:** iLiNS study locations and selected aspects of study designs^a^

Trial short name	Design and location	Intervention arms	As grouped for this analysis	Sample size for this analysis	Energy and nutrient content of supplements^b^	Ages & duration of infant supplementation
Burkina ZINC	Community‐based cluster‐randomized trial Dandé Health District, southwestern Burkina Faso	No LNS^c^ 20 g LNS Four groups with varying amounts of zinc	No LNS 20 g LNS	665 1957	‐‐ 117 kcal 22 micronutrients 2.5 g protein 9.5 g fat	9–18 months of age Duration: 9 months
Malawi DOSE	Community‐based individually randomized controlled single‐blind trial Mangochi District, southern Malawi	No LNS^c^ 10 g LNS 20 g LNS, no milk 20 g LNS with milk 40 g LNS, no milk 40 g LNS with milk	No LNS 10 g LNS 20 g LNS 40 g LNS	227 199 443 449	‐‐ 55 kcal 117 kcal 241 kcal All LNS provided 22 micronutrients (equal amounts across all LNS), 1–5 g protein 4.7–18.9 g fat	6–18 months of age Duration: 12 months
DYAD‐Ghana	Clinic‐based individually randomized controlled trial Yilo Krobo and Lower Manya Krobo Districts, Eastern Region, Ghana	IFA group, no LNS MMN group, no LNS LNS group, 20 g LNS	No LNS 20 g LNS No LNS 20 g LNS No LNS 20 g LNS	733 360	Pregnancy through 6 months post‐partum: IFA: 60 mg iron and 400 μg folic acid MMN: 20 mg iron, 400 μg folic acid and 16 other micronutrients LNS: 118 kcal, 2.6 g protein, 10 g fat, 20 mg iron, 400 μg folic acid and 20 other micronutrients Infant: As above for ZINC	6–18 months of age Duration: 12 months
DYAD‐Malawi	Design as for DYAD Ghana Mangochi District, southern Malawi			418 207		

a LNS = lipid‐based nutrient supplement(s); IFA = iron and folic acid; MMN = multiple‐micronutrient. For DYAD trials, the IFA group received IFA during pregnancy, a placebo for 6 months postpartum, and infants were not supplemented; the MMN capsule group received MMN during pregnancy and for 6 months postpartum, and infants were not supplemented; and the LNS group received 20 g LNS during pregnancy and for 6 months postpartum, and infants received a different 20 g LNS from 6 to 18 months of age.

b See Arimond et al. (2015) for more detailed description of the nutrient content of the LNS and Adu‐Afarwuah et al. (2015) for detailed description of the MMN supplements provided in the DYAD trials.

c Groups receiving no LNS during the intervention period received a delayed intervention.

One trial in Malawi (hereafter, “Malawi DOSE”) was a community‐based individually randomized controlled clinical trial to evaluate outcomes in five LNS groups compared with infants who received a delayed intervention. The LNS groups varied in quantity (“dose” of 10, 20, or 40 g) and in milk content at two dosage levels (20 and 40 g; Maleta et al., 2015). For the purposes of the present analysis, four comparison groups were considered, receiving 0, 10, 20, or 40 g of LNS (i.e., we collapsed groups receiving milk‐containing and non‐milk‐containing LNS at 20 and 40 g). Infants were recruited at 6 months of age, and those in intervention groups were supplemented until 18 months of age.

Two sister trials in Ghana and Malawi (hereafter, “DYAD‐Ghana” and “DYAD‐Malawi”) were designed to evaluate the efficacy of SQ‐LNS given to women during pregnancy and the first 6 months postpartum as well as to their offspring from 6 to 18 months of age. The DYAD trials were individually randomized controlled trials with three equal‐size groups receiving (a) the local standard of care supplement during pregnancy (iron and folic acid) and a placebo postpartum for the mother (IFA group), (b) a multiple‐micronutrient capsule for the mother during pregnancy and the first 6 months postpartum (MMN group), or (c) 20 g SQ‐LNS for the mother during pregnancy and the first 6 months postpartum and 20 g SQ‐LNS for infants from 6 to 18 months (SQ‐LNS group; Adu‐Afarwuah et al., 2015; Ashorn et al., 2015a; Ashorn et al., 2015b). For the purposes of this analysis, the two groups that did not receive infant SQ‐LNS (i.e., the IFA and MMN groups) were combined and compared with the SQ‐LNS group.

In the Burkina ZINC and Malawi DOSE trials, age‐eligible infants were identified and recruited based on community censuses. Recruitment into the two DYAD trials was from clinics and hospitals where women sought antenatal care. In all trials, it was not possible to completely blind participants and certain field staff; that is, participants as well as field staff distributing LNS knew who received LNS, tablets, or no supplement. However, all data analysts were blinded to group assignment until first analysis of outcomes was complete.

### 2.2 Study sites and participants

In both Burkina ZINC and Malawi DOSE, exclusion criteria included low weight‐for‐height, edema, severe anemia, severe illness warranting hospital referral, history of peanut allergy, history of anaphylaxis or serious allergic reaction to any substance, or concurrent participation in another clinical trial. In addition to age (9.0–9.9 months in Burkina Faso and 5.5–6.5 months in Malawi), inclusion criteria included residence in the study area with no plans to move and signed informed consent from at least one guardian. The Burkina ZINC trial was conducted from April 2010 to July 2012 in rural communities of the Dandé Health District in southwestern Burkina Faso. The Malawi DOSE trial was conducted from November 2009 to May 2012 in rural and semi‐urban areas in Mangochi District in southern Malawi.

The sister DYAD trials in Ghana and Malawi recruited women in the first half of pregnancy (<20 weeks gestation) who resided in the study area, with no plans to move and who gave informed consent. Age eligibility differed by site and was at least 18 years in Ghana but at least 15 years in Malawi. Women known to be HIV‐positive were excluded in Ghana (based on examination of the antenatal card) but not in Malawi. Both sites excluded women with severe health problems identified at enrollment; allergy toward peanut (both sites) or milk (Ghana only); history of asthma, anaphylaxis, or serious allergic reaction to any substance; or concurrent participation in another clinical trial or previous participation in the DYAD trial (i.e., during a previous study pregnancy carried to term).

In Ghana, the study site was the Somanya‐Kpong area, about 70 km north of the capital, Accra, in the Yilo Krobo and Lower Manya Krobo Districts. Enrollment was from two hospitals, a polyclinic and a clinic, from December 2009 through December 2011. In Malawi, enrollment took place from February 2011 to August 2012 in two hospitals (Mangochi and Malindi) and two rural health centers (Lungwena and Namwera) in Mangochi District, southern Malawi.

### 2.3 Interventions

Supplements and interventions have been described in detail previously (Arimond et al., 2015; Ashorn et al., 2015a; Adu‐Afarwuah et al., 2015b; Hess et al., 2015; Maleta et al., 2015). Table 1 summarizes the nutrient content and duration of supplementation for each trial. All supplements were designed to be consumed daily. For infants, mothers were advised to mix the LNS with foods such as porridge and to give one‐half the LNS dose at two time points during the day.

In all trials, supplements were delivered to participants' homes on a weekly or biweekly basis. During these visits, morbidity surveillance and referral or treatment was provided, with the following differences among sites: in DYAD‐Ghana and both Malawi trials, all groups, including control or delayed intervention groups, received surveillance and referral. In Burkina ZINC only, the intervention group received immediate treatment by study personnel for uncomplicated diarrhea, fever, or malaria and referral for more serious illness, whereas the non‐intervention group received no disease surveillance or referrals, and there was no contact between study personnel and non‐intervention participants between baseline and endline data collection.

The trials described here were not designed to include an educational or behavior change component related to IYCF. However, certain brief didactic nutrition messages—consistent with local Ministry of Health messages—were conveyed at enrollment and infrequently thereafter to help ensure that the LNS intervention did not negatively affect breastfeeding or diversification of infant diets. In two sites, when infants presented at interim study visits with low weight‐for‐height, messages were repeated by study anthropometrists (Burkina ZINC; with measurement at 12‐ and 15‐month visits for intervention groups only) or feeding advice was provided by study nurses (DYAD‐Ghana, 6.5% of infants).

Core nutrition messages were identified for use in all trials, with cultural and linguistic adaptation for each site. The core messages were (a) Breastfeed your child just as you did before you were part of the study and (b) Give your child fruits, vegetables, meat, fish, eggs and dairy products as often as you can.

### 2.4 Outcomes and covariates

Infant and young child feeding practices were secondary outcomes for the four trials. All outcomes were assessed at 9, 12, 15, and 18 months of age (Malawi DOSE and both DYAD trials) or 18 months of age only (Burkina ZINC) based on caregiver (usually maternal) recall of practices in response to structured survey questions using instruments adapted from a model questionnaire from the WHO (2010). We report here only on outcomes at 18 months, the time point at which we have data from all four trials. Dietary diversity and consumption of micronutrient‐rich food groups were assessed through a guided free recall of liquids and foods consumed by the child yesterday and a list‐based recall of the number of days food groups were consumed in the 7 days preceding the interview. The LNS was not “counted” in any food group, so that impact on infant feeding practices exclusive of LNS could be assessed.

We defined ten IYCF practice outcomes:

1. Infant still breastfed at 18 months (%)
2. Infant breastfed at least six times yesterday during the day (%)
3. Met WHO criterion for frequency of complementary feeding yesterday (three or more times for breastfed and four or more times for non‐breastfed infants; %; data from two trials only)
4. Met WHO criterion for food group diversity yesterday (four or more out of seven defined groups: grains, roots, and tubers; legumes and nuts; dairy products; flesh foods; eggs; vitamin A‐rich fruits and vegetables; other fruits and vegetables; %)
5. Number of animal‐source food groups yesterday (range 0–5; summing organ meats, other meat/poultry, fish, eggs, dairy)
6. Number of fruit/vegetable food groups yesterday (range 0–5; summing vitamin A‐rich orange/yellow vegetables, dark green leafy vegetables, other vegetables, vitamin A‐rich fruits, other fruits)
7. Animal‐source food group score for last 7 days (range 0–28; the score sums the number of days the infant consumed foods from each of four animal‐source food groups over 7 days; groups are similar to those for yesterday, but organ meats and other flesh foods were grouped together)
8. Fruit/vegetable food group score for last 7 days (range 0–35; score sums the number of days the infant consumed foods from each of five groups over 7 days; groups are the same as those for yesterday)
9. In lowest tertile of animal‐source food group score for last 7 days (%), with tertiles defined within each site
10. In lowest tertile of fruit/vegetable food group score for last 7 days (%), with tertiles defined within each site.

In addition to WHO indicators, we constructed a number of other indicators. There is no standard approach to measurement of frequency of breastfeeding, and precise recall of frequency is difficult for women who breastfeed on demand, as is common in our study areas. We presented women with five response options for frequency (1. Not at all; 2. Only at night; 3. Very little, only 1 or 2 times during the day; 4. Moderately, about 3 to 5 times during the day; 5. Very often, at least 6 times during the day) and asked them to select the response closest to their recalled practice yesterday. We considered any response other than the last to be a proxy for reduced frequency of breastfeeding. Because the WHO indicator for dietary diversity is not very sensitive, we constructed a number of additional indicators related to food group diversity, including counts for fruits and vegetables and animal‐source foods yesterday and last week, and outcomes assessing the prevalence of low diversity over the past 7 days. The overall aim of measurement of this set of diversity indicators was to allow detection of any changes in feeding practices, should they have occurred.

Covariate data were also collected using standard data collection tools and methods. Covariates were selected based on a conceptual model identifying household, maternal, and infant characteristics that could theoretically affect infant feeding practices independently of the intervention.

Baseline length‐for‐age and weight‐for‐length were measured in duplicate or triplicate by standardized anthropometrists; *z*‐scores (length‐for‐age z‐score (LAZ) and weight‐for‐length z‐score (WLZ)) were assessed as covariates in the Burkina ZINC and Malawi DOSE trials only, because in the DYAD trials, baseline was antenatal. In all trials, child sex and age in days at endline were assessed as covariates. In all trials, household‐level baseline covariates comprised distance in meters to the nearest weekly market; an asset score derived from a principal components analysis (Vyas & Kumaranayake, 2006); a small livestock score measured in tropical livestock units (FAO, 2003); household food insecurity (Coates, Swindale, & Bilinsky, 2007); and number of children aged 5 and under in the household (proxied by parity in one site). Maternal baseline characteristics comprised age, body mass index, education level, ethnicity or main language spoken, marital status, and maternal HIV status (DYAD‐Malawi only). Season at endline was included as a concurrent covariate, as this could affect food groups consumed. In addition to the household food insecurity score, for descriptive purposes, we calculated a household hunger score, considered to be more valid across cultures (Ballard, Coates, Swindale, & Deitchler, 2011).

### 2.5 Sample size

For all trials, sample sizes were selected based on primary outcomes. For the secondary outcomes reported here, we calculated statistical power post hoc based on analysis sample sizes (i.e., those children remaining in the trial at endline and with data for both outcomes and covariates); analysis sample sizes are detailed in Table 1 and Supplemental Figures 1–4. For the Burkina ZINC trial, our analysis includes 2622 children. For the Malawi DOSE trial, our analysis includes 1318 children. The 20 and 40 g groups are larger (Table 1) because milk‐ and non‐milk‐containing LNS groups were combined for those dosages. For the DYAD‐Ghana and DYAD‐Malawi trials, our analysis includes 1093 and 625 children, respectively. For count variables (number of food groups yesterday and scores for last week), we had 80% power to detect differences ranging from 0.1 to 0.3 food groups yesterday and from 0.6 to 1.8 points for the scores for last week (*P* < 0.05). For dichotomous outcomes, power depends on prevalence, as well as on sample size. For the Burkina ZINC trial, we had 80% power to detect differences ranging from 2 to 8 percentage points across the dichotomous outcomes. For the Malawi DOSE, Ghana‐DYAD, and Malawi‐DYAD trials, we could detect percentage point differences of 10–16, 8–9, and 6–12, respectively.

### 2.6 Statistical analysis

All analyses were done using SAS version 9.3 (SAS Inst. Cary, NC, USA) or Stata version 13.1 (StataCorp, TX, USA). Analysis of the effect of the interventions was on an intention‐to‐treat basis, and analysis was performed within, not across, trials. A statistical analysis plan was published at our website (http://iLiNS.org) prior to analysis.

Differential attrition was assessed. Baseline characteristics were compared between those lost to follow‐up and those in the analysis sample, using simple bivariate tests. Baseline characteristics were also compared between intervention groups in the analysis sample for descriptive purposes, but following Consolidated Standards of Reporting Trials (CONSORT) guidelines, statistical tests of differences are not reported. In each site, we assessed prespecified covariates described above for their relationship to each of the 10 outcomes. All covariates significantly related to an outcome (*P* < 0.10) were included in multivariate models for that outcome. In the three individually randomized trials, we tested the null hypothesis of no difference among groups using analysis of variance or logistic regression, with and without controlling for significant covariates. In the cluster‐randomized Burkina ZINC trial, we analyzed mixed models (SAS proc mixed) for continuous and logistic models for dichotomous outcomes (SAS proc glimmix). Models were adjusted for random effects of the village and the household. In the Malawi DOSE trial, when the global null hypothesis was rejected at 0.05 level for any outcome, we performed post hoc pairwise comparisons using appropriate adjustments for multiple comparisons.

### 2.7 Ethical review and registration

The Burkina ZINC trial was approved by the Institutional Review Boards of the Centre Muraz in Bobo‐Dioulasso and the University of California, Davis. The DYAD‐Ghana trial was approved by the Institutional Review Boards of the University of California, Davis; the Ghana Health Service; and the University of Ghana Noguchi Memorial Institute for Medical Research. The Malawi DOSE and DYAD‐Malawi trials were approved by the University of Malawi College of Medicine Research and Ethics Committee and the ethics committee of Pirkanmaa Hospital District, Finland. The trials were registered at http://www.ClinicalTrials.gov as NCT00944281 (Burkina Faso), NCT00970866 (DYAD‐Ghana), NCT00945698 (Malawi DOSE), and NCT01239693 (DYAD‐Malawi).

## 3 RESULTS

### 3.1 Attrition and baseline characteristics

Attrition did not vary by intervention group in any of the trials. Attrition was lower in Burkina ZINC and DYAD‐Ghana (17–19%) and higher in the two Malawi trials (28–32%) as shown in Supplemental Figures 1–4. Comparing baseline characteristics of those lost to follow‐up with those in the analysis samples, there were very few differences in Burkina ZINC and DYAD‐Ghana, and even when significant, differences were generally small. In Malawi, and particularly in the DYAD‐Malawi trial, differences were larger and more systematic, with those lost to follow‐up living closer to towns and differing on a number of characteristics related to this; see Supplemental Table 1 for details.

**Table** 2 provides descriptive statistics for analysis samples for baseline characteristics and the two endline covariates (child age and season) and shows contrasts between the trial sites. Supplemental Tables 2–5 show the same characteristics by intervention group, for each trial.

**Table 2:** Comparison of selected baseline characteristics and two concurrent covariates, by iLiNS trial^a^

		**ZINC**		**DOSE**		**DYAD‐G**		**DYAD‐M**	
		n=2622		n=1318		n=1093		n=625	
		Mean/%	SD	Mean/%	SD	Mean/%	SD	Mean/%	SD
**Season of interview**	% rainy season	37.6		48.9		50.7		41.3	
**Distance to market (m)**	Mean	1699	1800	3257	2787	1900	1830	18578	12148
**Small livestock score (TLU)** b	% with none	8.1		59.5		71.6		35.5	
	Mean TLU score	1.01	1.46	0.070	0.183	0.165	0.544	0.225	0.445
**Household hunger score (%)**	% scoring >= 2^c^	3.5		26.5		3.3		18.3	
	Mean score	0.13	0.52	0.95	1.22	0.160	0.523	0.67	1.00
**Focus child is only child under 5 y (%)** d	%	33.2		44.5		56.3		n/a	
**Nulliparous at baseline**	%	n/a		n/a		n/a		22.7	
**HIV positive at baseline**	%	n/a		n/a		n/a		11.9	
**Maternal education (y)**	% with none	59.6		21.9		8.5		29.0	
	Mean number of y	n/a		4.5	3.6	7.7	3.6	3.6	3.4
**Maternal age (y)**		27.2	7.7	26.5	6.5	26.7	5.4	25.3	6.0
**Maternal BMI (kg/m^2^)**		20.8	2.5	21.9	2.9	24.9	4.7	21.5	2.6
**Marital status (%)**	In informal union	n/a		n/a		59.8		n/a	
	Single/divorced/widowed	2.1		12.0		1.1		10.5	
	Married, monogamous husband	53.9		43.5		39.1		64.4	
	Married, polygamous husband	44.0		44.5		n/a		25.1	
**Child sex**	% male	50.6		50.1		48.3		47.8	
**Child age at interview (days)**	Mean	558	12	548	13	550	4	549	2
**Weight‐for‐length z‐score** e	Mean	‐1.00	1.05	0.29	1.10	n/a		n/a	
**Length‐for‐age z‐score** e	Mean	‐1.21	1.10	‐1.38	1.04	n/a		n/a	

a ZINC is the iLiNS‐ZINC trial in Burkina Faso; DOSE is the iLiNS DOSE trial in Malawi; DYAD‐G is the iLiNS DYAD trial in Ghana; DYAD‐M is the iLiNS DYAD trial in Malawi. Cells are marked “n/a” if a variable or response category was not available or not relevant or not used in the analysis in the site.

b TLU = Tropical livestock units. TLU are a standardized animal unit calculated by generating a weighted sum of the number of animals owned, where the weights are determined by “feeding requirement” (FAO 2003). Small livestock include sheep, goats, pigs, chickens, and rabbits.

c The Household Hunger Score is considered more valid for cross‐cultural use than are other experiential food security measures. It is based on three questions related to hunger, and a score of ≥2 indicates moderate to severe hunger in the household (Ballard et al. 2011).

d In DYAD‐Ghana, this is the percent who reported no children under 5 years of age in the household at enrollment (antenatally).

e Weight‐for‐length and length‐for‐age z‐scores are from baseline for DOSE and ZINC. Mean age at baseline in DOSE was 179 d (5.9 mo) and in ZINC was 287 d (9.4 mo). Infant z‐scores were not used as covariates in the DYAD trials because they could have been impacted by the antenatal intervention.

Comparing across trials (Table 2), on average, women in the DYAD‐Ghana site had more education and markedly higher body mass index, and households were less likely to own small livestock, reflecting the more urban nature of this study site. In both DYAD‐Ghana and Burkina ZINC, households were relatively close to markets and were very unlikely to report recently experiencing moderate to severe hunger. Households in both Malawi trials were situated farther from main markets (especially in the DYAD‐Malawi trial) and were more likely to report recently experiencing moderate to severe hunger. Households in Burkina ZINC were most likely to report small livestock ownership. However, women in Burkina ZINC had the least education. Child age at endline was very similar across sites. At baseline, WLZ was lower in Burkina ZINC than in Malawi DOSE (−1.00 vs. +0.29, respectively).

### 3.2 Effect of interventions on infant and young child feeding practices

**Figure 1:**
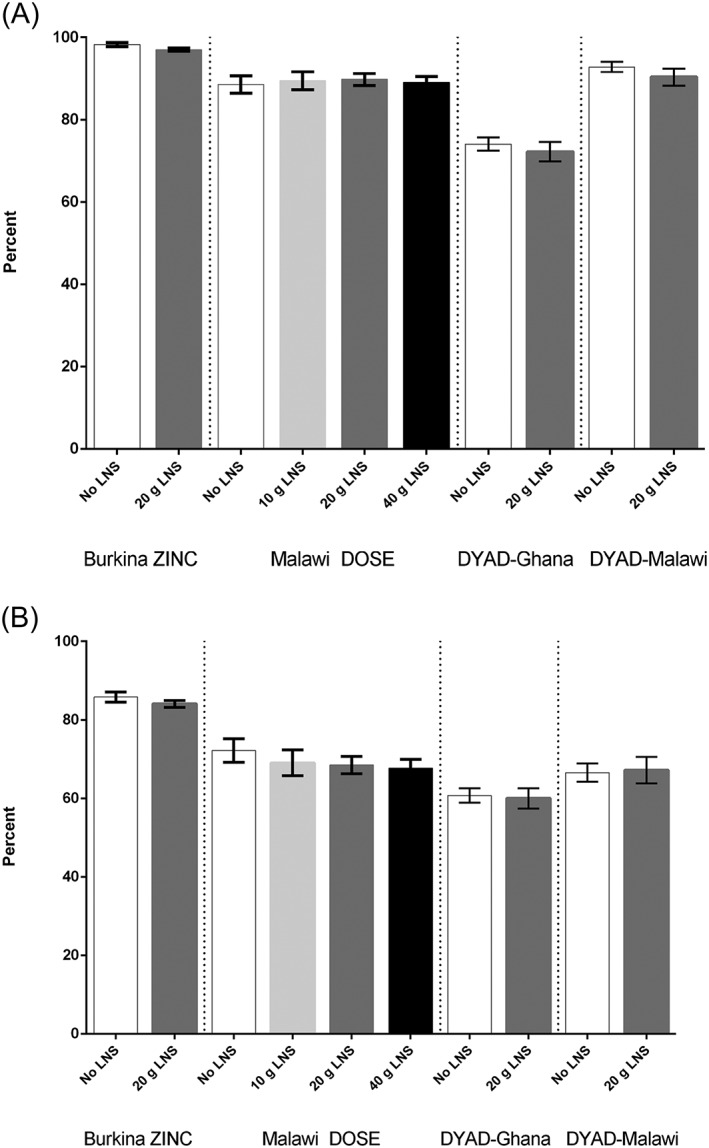
(a) Continued breastfeeding at 18 months of age, by trial and intervention group**.** (b) Frequent breastfeeding (six or more times yesterday during the day) at 18 months of age, by trial and intervention group

Figure 1 shows maternal report of endline breastfeeding practices by intervention group in all trials; there were no differences between intervention groups in any of the four trials. The prevalence of continued breastfeeding ranged from 74% in the Ghana site to 97% in Burkina Faso, and the pattern for prevalence of frequent breastfeeding (six or more times yesterday during the day) was similar.

**Figure 2:**
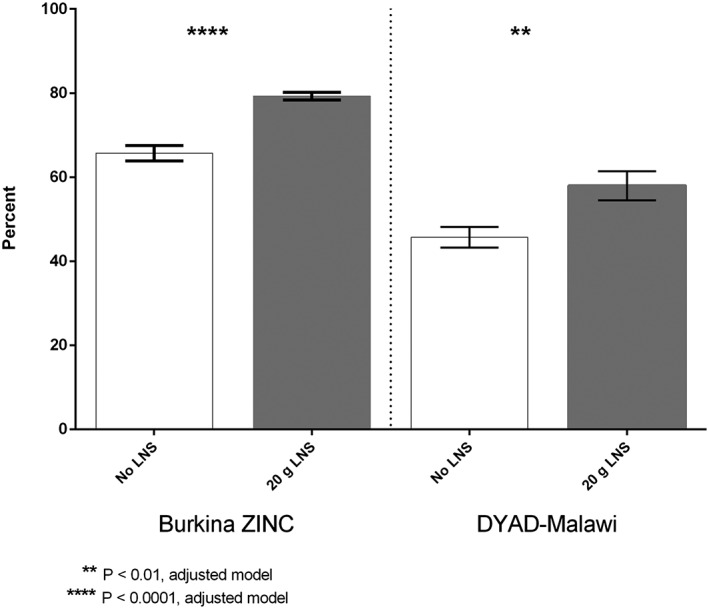
Prevalence of meeting recommended the World Health Organization frequency of feeding yesterday at 18 months of age, by trial and intervention group

**Figure** 2 shows the percentage of children reported to meet the WHO criterion for frequency of feeding in two trials; data on frequency of feeding were not available for the other two trials. In both the Burkina ZINC trial and the DYAD‐Malawi trial, children in the SQ‐LNS intervention groups were more likely to meet the criterion compared with the groups receiving no SQ‐LNS. The percentage point difference between groups receiving and not receiving SQ‐LNS was similar in the two trials, at 12–14% (*P* < 0.0001 in Burkina and *P* = 0.005 in Malawi). In the DYAD‐Malawi trial where we had additional data on staple food consumption, children in the SQ‐LNS group were also reported to consume staple foods (*nsima* and porridge) more frequently the previous day (*P* = 0.005; results not shown).

**Table** 3 provides results for the remaining seven outcomes, all related to maternal report of infant dietary diversity. The table presents unadjusted percents and means and *P*‐values both for unadjusted models and for models adjusted for all covariates that were significantly related to the outcome. Supplemental Table 6 lists the covariates adjusted for in models of each outcome for all trials. For almost all outcomes, covariate adjustment did not change results of significance tests.

**Table 3:** Food group diversity at 18 months of age by trial and intervention group, unadjusted proportions or means, unadjusted and adjusted P‐values^a^

	Percent or mean	SD	Percent or mean	SD					Unadjusted p‐value^b^	Adjusted p‐value^c^
**Burkina ZINC**	**No LNS (n=665)**		**20 g LNS (n=1957)**							
4 or more food groups yesterday (WHO indicator, %)^d^	31.0		39.3						0.182	0.399
Number of ASF food groups yesterday, range 0‐5^e^	0.51	0.74	0.61	0.78					0.216	0.334
Number of fruit/vegetable food groups yesterday, range 0‐5^f^	1.70	0.87	1.91	0.92					0.019	0.024
ASF score last 7 d, range 0‐28^g^	5.60	3.73	7.17	3.65					0.058	0.071
Fruit/vegetable score last 7 d, range 0‐35^h^	10.1	4.6	11.1	4.6					0.071	0.082
Lowest tertile for 7 d ASF score (score of 0–6, %)	56.6		37.4						0.023	0.020
Lowest tertile for 7 d fruit/vegetable score (score of 0–8, %)	41.7		33.9						0.106	0.007
**Malawi DOSE**	**No LNS (n=227)**		**10 g LNS (n=199)**		**20 g LNS (n=443)**		**40 g LNS (n=449)**			
4 or more food groups yesterday (WHO indicator, %)^d^	63.8		68.3		69.0		69.4		0.497	0.641
Number of ASF food groups yesterday, range 0‐5^e^	1.01	0.87	1.04	0.86	0.97	0.79	1.00	0.84	0.765	0.985
Number of fruit/vegetable food grps yesterday, range 0‐5^f^	2.02	1.04	2.15	1.01	2.21	1.04	2.13	1.00	0.165	0.043^i^
ASF score last 7 d, range 0‐28^g^	5.88	3.80	5.83	3.93	5.98	4.02	5.48	3.54	0.240	0.064
Fruit/vegetable score last 7 d, range 0‐35^h^	12.8	5.3	13.3	5.7	12.8	5.5	13.1	5.4	0.653	0.706
Lowest tertile for 7 d ASF score (score of 0–4, %)	41.3		42.2		42.3		44.3		0.881	0.655
Lowest tertile for 7 d fruit/vegetable score (0–11, %)	42.0		37.2		41.7		38.0		0.516	0.680
**DYAD‐Ghana**	**No LNS (n=733)**		**20 g LNS (n=360)**							
4 or more food groups yesterday (WHO indicator, %)^d^	77.0		72.8						0.123	0.191
Number of ASF food groups yesterday, range 0‐5^e^	2.08	0.99	2.02	0.96					0.291	0.493
Number of fruit/vegetable food groups yesterday, range 0‐5^f^	1.47	0.75	1.48	0.84					0.824	0.719
ASF score last 7 d, range 0‐28^g^	11.6	5.0	11.3	4.9					0.472	0.462
Fruit/vegetable score last 7 d, range 0‐35^h^	10.9	4.4	10.9	4.2					0.955	0.908
Lowest tertile for 7 d ASF score (score of 0–9, %)	37.1		36.4						0.833	0.671
Lowest tertile for 7 d fruit/vegetable score (score of 0–9, %)	42.1		39.3						0.368	0.393
**DYAD‐Malawi**	**No LNS (n=418)**		**20 g LNS (n=207)**							
4 or more food groups yesterday (WHO indicator, %)^d^	50.1		56.2						0.159	0.164
Number of ASF food groups yesterday, range 0‐5^e^	0.94	0.84	0.96	0.80					0.843	0.868
Number of fruit/vegetable food groups yesterday, range 0‐5^f^	1.78	1.02	1.84	1.03					0.502	0.470
ASF score last 7 d, range 0‐28^g^	5.4	3.5	5.9	3.7					0.076	0.100
Fruit/vegetable score last 7 d, range 0‐35^h^	11.2	5.4	11.4	5.0					0.648	0.642
Lowest tertile for 7 d ASF score (score of 0–4, %)	47.5		38.4						0.035	0.038
Lowest tertile for 7 d fruit/vegetable score (score of 0–9, %)	36.3		35.0						0.753	0.883

a LNS=lipid‐based nutrient supplement; ASF=animal‐source food.

b For Burkina ZINC (cluster randomized): P‐values from mixed models (proc mixed) for continuous variables and logistic models for dichotomous (proc glimmix). Models adjusted for the random effect of the village and the concession/compound when possible. For all other sites (individually randomized trials): P‐values from ANOVA, and LOGIT models for dichotomous outcomes.

c Models adjusted for all covariates that were significant in bivariate models (see Supplemental Table 6 for lists of covariates controlled for, for each outcome in all sites).

d At least 4 out of the following 7 food groups: grains, roots and tubers; legumes and nuts; dairy products; flesh foods; eggs; vitamin A rich fruit and vegetables; other fruits and vegetables.

e The 5 ASF groups are: 1) organ meats; 2) other meat/poultry; 3) fish; 4) eggs; and 5) dairy.

f The 5 fruit and vegetables groups are: 1) vitamin A‐rich orange/yellow vegetables; 2) dark green leafy vegetables; 3) other vegetables; 4) vitamin A‐rich fruits; and 5) other fruits.

g Score sums four groups over seven days; groups are similar to those for yesterday, but organ meats and other flesh foods are grouped together.

h Score sums five groups over seven days; groups are the same as those for yesterday.

i In post‐hoc pairwise comparisons, the only significant difference was between the 0 g and the 20 g group.

The prevalence of meeting the WHO criterion for food group diversity ranged from 37% in the Burkina Faso site to 76% in the Ghana site and did not differ by intervention group in any site. There were no significant between‐group differences in any of the food group diversity outcomes in the DYAD‐Ghana trial. In the Malawi DOSE trial, there was one between‐group difference, in the number of fruit/vegetable groups reported to be consumed the previous day between the group receiving no LNS (2.0 of five fruit/vegetable groups yesterday) and the group receiving 20 g LNS (2.2 of five groups; *P* = 0.04). In the two trials where frequency of feeding differed by intervention group—the Burkina ZINC trial and the DYAD‐Malawi trial—there were also some differences in food group diversity outcomes. In Burkina ZINC, children who received SQ‐LNS were reported to consume slightly more diverse fruits and vegetables the previous day compared with children that did not (1.9 vs. 1.7 groups, respectively; *P* = 0.02) and were less likely to be in the lowest tertile for both the animal‐source food score and the fruit/vegetable score for the last 7 days (*P* = 0.02 and *P* = 0.007, respectively, with the percentage point differences being 19% and 8%, respectively). Similarly, in the DYAD‐Malawi trial, fewer children in the SQ‐LNS group were in the lowest tertile for the 7‐day animal‐source food score (*P* = 0.04), though the percentage point difference (9%) was lower than in Burkina ZINC.

## 4 DISCUSSION

In four supplementation trials in diverse sites in Africa, provision of 10–40 g of LNS for daily consumption by infants did not decrease the prevalence of continued breastfeeding or the frequency of breastfeeding at 18 months of age, after 9–12 months of supplementation. Overall, there was little impact on IYCF practices at 18 months, but there was evidence from two of the four trials that frequency of feeding may have been positively impacted; these were the only two trials with data on frequency of feeding. In the same two trials, there was evidence that animal‐source food scores for the past week were less likely to be low among children who had received SQ‐LNS, compared with those with no SQ‐LNS.

Concerning our results for frequency of feeding, in the DYAD‐Malawi trial, there is suggestive evidence that provision of the SQ‐LNS may have increased frequency of feeding with staple foods; this could have been related to the intervention if caregivers were adding SQ‐LNS to porridge twice a day as advised. However, in the Burkina ZINC trial, a substudy employing direct observation methods documented that at 16 months of age, most infants (86%) consumed the SQ‐LNS “as is” and not mixed with food (Abbeddou et al., 2015). The recall question on frequency of feeding did not distinguish between meals and snacks. Thus, the increase in frequency of feeding reported by mothers may have reflected extra snacks of LNS.

At 18 months of age, the transition from breastfeeding to family food is largely complete; it is possible that provision of supplements could impact practices earlier in the transition. Preliminary analysis of outcomes at 9, 12, and 15 months of age is consistent with our endline analysis; that is, there were no impacts on practices in the Malawi DOSE trial or the DYAD‐Ghana trial, while several indicators at each time point suggested improved dietary diversity or frequency of feeding in DYAD‐Malawi (results not shown). Data were not collected at interim time points in the Burkina ZINC trial.

Previously, a handful of studies have assessed the impact of provision of LNS on breastfeeding, complementary food intake, and feeding practices. Two quantitative studies showed no difference in breast milk intake after 1–4 months of supplementation with 10–50 g LNS, compared with fortified blended food (Galpin et al., 2007) or compared with no supplement (Kumwenda et al., 2014).

Energy intake from complementary foods among children given or not given LNS was assessed in several studies. In a previous randomized trial in southern Malawi, infants receiving 43 g of LNS for 9 weeks had higher energy intakes from complementary foods than non‐supplemented infants. When the LNS was excluded, energy intakes did not differ (Thakwalakwa et al., 2015). In our DOSE trial in southern Malawi, we assessed energy intakes at 9–10 months of age, after 3–4 months of supplementation with 0–40 g of LNS. As in the previous trial, energy intakes were higher in the supplemented groups but did not differ from the control (0 g) group when LNS was excluded from the analysis (Hemsworth et al., 2016). Both authors concluded that LNS in the doses consumed did not displace local foods.

In a previous study in Ghana, energy intake was assessed each month from 7 to 12 months of age during a randomized trial of supplementation with 20 g SQ‐LNS or multiple micronutrients (Adu‐Afarwuah et al., 2007). As in the Malawi studies, energy intakes were higher in the SQ‐LNS group but did not differ when energy from SQ‐LNS was excluded.

In a cluster‐randomized intervention trial in Honduras, children aged 6–18 months at enrollment received 46–70 g per day of LNS (with dosage depending on age) for 12 months or no LNS, and dietary intake was measured at baseline and every 3 months thereafter. The change in energy intake from baseline to each time point was higher in the LNS group, and the authors concluded that LNS did not displace local foods (Flax, Siega‐Riz, Reinhart, & Bentley, 2015). This is the only study we are aware of that assessed the impact of LNS on energy intakes at the end of a longer (12 month) period of supplementation.

In the Malawi DOSE trial (Hemsworth et al., 2016) and in the study in Honduras (Flax et al., 2015), food group intake was also examined. In Malawi DOSE, there was no difference in food group intakes except for the comparison of the 40 g LNS group with the control (0 g), where there was lower intake of legumes in the LNS group. Similarly in the Honduras study, the only food groups that differed were those associated with the LNS itself, where intake was higher (e.g., of nuts and nut butters).

Few previous studies have looked at IYCF practices. Two small studies in Malawi employed structured observations before and at multiple time points during 12 weeks of supplementation with LNS (Flax et al., 2008) or comparing an LNS group and a group receiving corn‐soy blend (Flax et al., 2010). The first study showed no difference in frequency of breastfeeding, in number of meals, or in total time spent feeding comparing before with during supplementation. The second showed no difference in the total number of feeding episodes or time spent feeding between the two types of supplement. One qualitative study in Haiti employing in‐depth interviews reported that some women increased frequency of breastfeeding while giving SQ‐LNS and others decreased frequency of breastfeeding (Lesorogol, Jean‐Louis, Green, & Iannotti, 2015). Lesorogol et al. also refer to a reduction in frequency of breastfeeding reflected in survey data for a group of infants who received SQ‐LNS for 6 months, but the survey data were not reported and the size of the reduction was not reported in either paper from this trial (Iannotti et al., 2014; Lesorogol et al., 2015). No differences in complementary feeding practices were reported.

In sum, our results are consistent with most previous studies showing no displacement of local foods and no impact on breastfeeding. Our results from two sites contrast with one previous observational study that showed no difference in frequency of complementary feeding after LNS was introduced (Flax et al., 2008); this could be due to differences in child age, duration of supplementation, and measurement method for frequency of feeding. Flax et al. (2008) enrolled 16 infants aged 6–17 months who were supplemented for 12 weeks and observed on four occasions. Our larger study assessed frequency of feeding at 18 months of age after 9–12 months of supplementation and according to caregiver recall, not by observation. In the second study cited above (Flax et al., 2010), there was no non‐intervention group, and infants receiving corn‐soy blend could also have been fed more frequently.

Strengths of our study include the randomized designs of all trials and the lack of differential attrition, as well as the harmonization across trials in the “information treatment” (delivery of brief nutrition messages) and in measurement of IYCF practices outcomes.

We recognize that substantial attrition, particularly in the two Malawi trials, limits generalizability of the results to populations, because those who were lost to follow‐up differed in certain characteristics from those included in these analyses. In both DYAD trials, sampling from clinics also restricts generalizability.

A second limitation is the potential for respondent bias inherent in all self‐reported data; more specifically, there could be potential for differential social desirability bias, particularly in the Burkina ZINC trial where the non‐intervention group had far less contact with the research team than did the intervention group. In the other three trials with weekly or biweekly visits to all participants, differential social desirability bias may be less likely but could still exist as an impact of provision of a “special food.”

A third limitation relates to relatively low statistical power for dichotomous outcomes, particularly for the Malawi and Ghana sites, which is a result of the trials having been powered for continuous primary outcomes. In comparing impact on outcomes between trials, we also note that statistical power varies, and therefore, we focus on the practical significance of the size of the differences in point estimates. We judge that the small but significant differences reported for several interval variables (food groups yesterday and scores for last week) are of little practical significance. However the differences in the proportion reported to meet the WHO criterion for frequency of feeding are of practical significance in the two sites where data were available. The differences in the proportion with low animal‐source food scores could also be meaningful.

We conclude that these four trials in diverse sites in Africa provide no evidence of negative impact of long‐term (9–12 months) provision of SQ‐LNS on IYCF practices at 18 months of age and provide some evidence that in certain settings, provision of LNS may positively impact frequency of feeding.

## SOURCE OF FUNDING

This publication is based on research funded in part by a grant to the University of California, Davis from the Bill & Melinda Gates Foundation, with additional funding from the Office of Health, Infectious Diseases, and Nutrition, Bureau for Global Health, U.S. Agency for.

International Development (USAID) under terms of Cooperative Agreement AID‐OAA‐A‐. 12–00005, through the Food and Nutrition Technical Assistance III Project (FANTA), managed by FHI 360. The findings and conclusions contained within are those of the authors and do not necessarily reflect positions or policies of the Bill & Melinda Gates Foundation.

## CONFLICTS OF INTEREST

The authors declare that they have no conflicts of interest.

## CONTRIBUTIONS

MA and KGD conceptualized the analyses and designed the data collection instruments. MA and SA conducted data analysis. SA, CK, HO, JH, EYJ and EO coordinated and supervised data collection.

AL, UA, SAA, SAV, SYH, and KGD designed and supervised the iLiNS trials. MA drafted the manuscript, and all authors critically commented on drafts and approved the final manuscript.

## Supporting information



Figure S1 Supporting info item
