# Supplementary material for: Impact of small quantity lipid‐based nutrient supplements on infant and young child feeding practices at 18 months of age: results from four randomized controlled trials in Africa
Source: Matern Child Nutr. 2016 Dec 2;13(3):e12377. doi: 10.1111/mcn.12377 (PMC5516197; doi:10.1111/mcn.12377)
Supplement: Supplementary file 1 — Figure S1 Supporting info item [file MCN-13-e12377-s001.doc]

**Supplemental Table 1. Comparison of analysis samples to those lost to follow-up – significant differencesa**

| **Characteristics** | **ZINC** |  | **DOSE** |  | **DYAD-G** |  | **DYAD-M** |  |
| --- | --- | --- | --- | --- | --- | --- | --- | --- |
|  | **Lost**  **(n=598** | **Analysis**  **(n=2622)** | **Lost**  **(n=614)** | **Analysis**  **(n=1318)** | **Lost**  **(n=227)** | **Analysis**  **(n=1093)** | **Lost**  **(n=244)** | **Analysis**  **(n=645)** |
| Distance to market (%) |  |  |  |  |  |  |  |  |
| Nearest quartile | --- | --- | 32.4 | 21.6 | --- | --- | 45.1 | 17.6 |
| Q2 | --- | --- | 25.6 | 24.7 | --- | --- | 25.3 | 24.8 |
| Q3 | --- | --- | 22.2 | 26.3 | --- | --- | 12.9 | 30.3 |
| Farthest quartile | --- | --- | 19.9 | 27.4 | --- | --- | 16.7 | 27.2 |
| Asset index (%) |  |  |  |  |  |  |  |  |
| Lowest quartile | --- | --- | 24.2 | 29.9 | --- | --- | 19.6 | 26.9 |
| Q2 | --- | --- | 19.0 | 22.7 | --- | --- | 20.1 | 26.5 |
| Q3 | --- | --- | 29.6 | 24.4 | --- | --- | 23.6 | 25.6 |
| Highest quartile | --- | --- | 27.2 | 23.0 | --- | --- | 36.7 | 21.0 |
| Small livestock score (% with none) | --- | --- | 67.8 | 59.7 | --- | --- | 53.0 | 35.5 |
| HFIA category (%) |  |  |  |  |  |  |  |  |
| Food secure | --- | --- | --- | --- | --- | --- | 29.2 | 15.8 |
| Mildly food insecure | --- | --- | --- | --- | --- | --- | 12.4 | 12.0 |
| Moderately food insecure | --- | --- | --- | --- | --- | --- | 26.7 | 32.2 |
| Severely food insecure | --- | --- | --- | --- | --- | --- | 31.7 | 40.1 |
| Number of other underfives | --- | --- | --- | --- | --- | --- | n/a | n/a |
| Nulliparous at baseline (%) | n/a | n/a | n/a | n/a | n/a | n/a | 36.2 | 22.7 |
| Maternal education (% with none) | --- | --- | 15.9 | 22.0 | --- | --- | 19.8 | 29.0 |
| Maternal age (mean (SD) in y) | 26.4 (7.7) | 27.2 (7.7) | 25.5 (5.8) | 26.5 (6.5) | --- | --- | 23.8 (6.0) | 25.3 (6.0) |
| Maternal BMI (mean (SD) kg/m2) | --- | --- | --- | --- | --- | --- | 22.0 (2.8) | 21.5 (2.6) |
| Maternal HIV status (% positive) | n/a | n/a | n/a | n/a | n/a | n/a | --- | --- |
|  |  |  |  |  |  |  |  |  |
|  |  |  |  |  |  |  |  |  |
|  |  |  |  |  |  |  |  |  |
| Ethnicity, Malawi (%) |  |  |  |  | n/a | n/a |  |  |
| Yao | n/a | n/a | --- | --- | n/a | n/a | 70.6 | 82.2 |
| Chewa | n/a | n/a | --- | --- | n/a | n/a | 16.2 | 10.7 |
| Other | n/a | n/a | --- | --- | n/a | n/a | 13.2 | 7.1 |
| Ethnicity, Burkina Faso (%) |  |  |  |  |  |  |  |  |
| Bobo | 18.8 | 23.3 | n/a | n/a | n/a | n/a | n/a | n/a |
| Mossi | 63.4 | 55.6 | n/a | n/a | n/a | n/a | n/a | n/a |
| Other | 14.5 | 14.2 | n/a | n/a | n/a | n/a | n/a | n/a |
| Language spoken in household, Ghana (%) |  |  |  |  |  |  |  |  |
| Krobo/Ga | n/a | n/a | n/a | n/a | 63.7 | 77.2 | n/a | n/a |
| Ewe | n/a | n/a | n/a | n/a | 16.3 | 11.9 | n/a | n/a |
| Other | n/a | n/a | n/a | n/a | 20.0 | 10.9 | n/a | n/a |
| Marital status (%) |  |  |  |  |  |  |  |  |
| Formally married | n/a | n/a | n/a | n/a | 30.4 | 39.1 | n/a | n/a |
| Single/divorced/widowed | --- | --- | --- | --- | n/a | n/a | 9.4 | 10.5 |
| Married, monogamous | --- | --- | --- | --- | n/a | n/a | 75.4 | 64.4 |
| Married, polygamous | --- | --- | --- | --- | n/a | n/a | 15.3 | 25.1 |
| Child sex (% male) | --- | --- | --- | --- | --- | --- | --- | --- |
| Child WLZ at baseline | --- | --- | --- | --- | n/a | n/a | n/a | n/a |
| Child LAZ at baseline | --- | --- | --- | --- | n/a | n/a | n/a | n/a |

^a^ ZINC is the iLiNS-ZINC trial in Burkina Faso; DOSE is the iLiNS DOSE trial in Malawi; DYAD-G is the iLiNS DYAD trial in Ghana; DYAD-M is the iLiNS DYAD trial in Malawi. WLZ=weight-for-length z-score; LAZ=length-for-age z-score. Cells with “---“ indicate no significant differences between the analysis sample and those lost to follow-up. “n/a” indicates the variable was not used for that trial. For filled cells, all P < 0.05 for bivariate tests (chi-squared or ANOVA).

**Supplemental Table 2. Burkina ZINC trial: Summary of baseline characteristics and two**

**concurrent covariates in groups in analysis sample receiving and not receiving LNSa**

| **Characteristics** | **No LNS**  **(n=665)** | **20 g LNS**  **(n=1957)** | **All**  **(n=2622)** |
| --- | --- | --- | --- |
| Season of interview |  |  |  |
| Rainy | 38.9 | 37.1 | 37.6 |
| Dry cool | 27.2 | 26.1 | 26.4 |
| Dry hot | 33.8 | 36.8 | 36.0 |
| Distance to market (%) |  |  |  |
| Nearest quartile | 34.5 | 21.9 | 25.1 |
| Q2 | 16.9 | 27.1 | 24.5 |
| Q3 | 19.7 | 27.3 | 25.4 |
| Farthest quartile | 28.8 | 23.6 | 24.9 |
| Asset index (%) |  |  |  |
| Lowest quartile | 38.1 | 20.3 | 24.8 |
| Q2 | 23.1 | 25.4 | 24.8 |
| Q3 | 21.1 | 26.4 | 25.1 |
| Highest quartile | 17.7 | 27.9 | 25.3 |
| Small livestock score (% with none) | 8.0 | 8.2 | 8.1 |
| HFIA category (%) |  |  |  |
| Food secure | 44.6 | 50.9 | 49.3 |
| Mildly food insecure | 18.9 | 16.8 | 17.3 |
| Moderately food insecure | 24.2 | 24.5 | 24.4 |
| Severely food insecure | 12.4 | 7.8 | 9.0 |
| Number of other underfives |  |  |  |
| One | 33.1 | 33.2 | 33.2 |
| Two | 38.4 | 40.7 | 40.1 |
| More than two | 28.5 | 26.1 | 26.7 |
| Maternal education (% with none) | 68.1 | 56.7 | 59.6 |
| Maternal age (mean (SD) in y) | 27.2 (7.6) | 27.3 (7.7) | 27.3 (7.7) |
| Maternal BMI (mean (SD) kg/m2) | 20.6 (2.4) | 20.9 (2.5) | 20.8 (2.5) |
| Ethnicity |  |  |  |
| Bobo | 38.3 | 18.1 | 23.3 |
| Mossi | 44.8 | 59.4 | 55.6 |
| Other | 9.9 | 15.8 | 14.2 |
| Marital status (%) |  |  |  |
| Single/divorced/widowed | 2.1 | 2.2 | 2.1 |
| Married, monogamous | 50.2 | 55.1 | 53.9 |
| Married, polygamous | 47.8 | 42.7 | 44.0 |
| Child sex (% male) | 50.4 | 50.7 | 50.6 |
| Child age at endline (mean (SD) in mo) | 18.4 (0.4) | 18.3 (0.4) | 18.3 (0.4) |
| Child WLZ at baseline (mean (SD)) | -1.05 (1.06) | -0.98 (1.04) | -1.00 (1.05) |
| Child LAZ at baseline (mean (SD)) | -1.19 (1.14) | -1.22 (1.09) | -1.21 (1.10) |

^a^ SQ-LNS=small quantity lipid-based nutrient supplements; WLZ=weight-for-length z-score; LAZ=length-for-age z-score.

**Supplemental Table 3. Malawi DOSE trial: Summary of baseline characteristics and two concurrent**

**covariates in groups in analysis sample receiving and not receiving LNSa**

| **Characteristics** | **No LNS**  **(n=227)** | **10 g LNS**  **(n=199)** | **20 g LNS**  **(n=443)** | **40 g LNS**  **(n=449)** | **All**  **(n=1318)** |
| --- | --- | --- | --- | --- | --- |
| Season of interview |  |  |  |  |  |
| Rainy | 50.9 | 48.5 | 47.3 | 49.6 | 48.9 |
| Harvest | 33.6 | 35.7 | 36.4 | 33.4 | 34.8 |
| Prepare land | 15.5 | 15.8 | 16.4 | 17.0 | 16.4 |
| Distance to market (%) |  |  |  |  |  |
| Nearest quartile | 24.7 | 24.6 | 19.3 | 20.1 | 21.3 |
| Q2 | 24.2 | 26.6 | 25.0 | 24.1 | 24.8 |
| Q3 | 22.5 | 18.6 | 29.3 | 29.0 | 26.4 |
| Farthest quartile | 28.6 | 30.2 | 26.4 | 26.8 | 27.5 |
| Asset index (%) |  |  |  |  |  |
| Lowest quartile | 29.4 | 22.3 | 30.8 | 32.9 | 30.0 |
| Q2 | 23.2 | 19.3 | 23.3 | 23.0 | 22.6 |
| Q3 | 22.2 | 28.9 | 24.4 | 23.8 | 24.5 |
| Highest quartile | 25.3 | 29.5 | 21.5 | 20.4 | 23.0 |
| Small livestock score (% with none) | 61.0 | 62.5 | 57.9 | 59.2 | 59.5 |
| HFIA category (%) |  |  |  |  |  |
| Food secure | 21.8 | 19.4 | 17.6 | 20.4 | 19.6 |
| Mildly food insecure | 6.4 | 3.6 | 8.0 | 8.8 | 7.3 |
| Moderately food insecure | 20.2 | 22.4 | 18.7 | 22.0 | 20.7 |
| Severely food insecure | 51.6 | 54.6 | 55.8 | 48.8 | 52.5 |
| Number of other underfives |  |  |  |  |  |
| One (focus child) | 41.3 | 46.2 | 48.4 | 41.6 | 44.5 |
| Two | 48.5 | 46.2 | 41.9 | 48.8 | 46.0 |
| Three - six | 10.2 | 7.7 | 9.6 | 9.6 | 9.4 |
| Maternal education (% with none) | 22.2 | 22.5 | 21.5 | 22.0 | 21.9 |
| Maternal age (mean (SD) in y) | 27.1 (7.1) | 25.9 (6.4) | 26.5 (6.6) | 26.5 (6.2) | 26.5 (6.5) |
| Maternal BMI (mean (SD) kg/m2) | 21.9 (2.7) | 21.8 (3.3) | 22.0 (2.7) | 22.0 (2.8) | 21.9 (2.9) |
| Ethnicity |  |  |  |  |  |
| Yao | 76.9 | 77.5 | 77.1 | 77.3 | 77.2 |
| Chewa | 15.4 | 18.3 | 20.0 | 16.9 | 17.9 |
| Other | 7.7 | 4.1 | 2.9 | 5.7 | 4.9 |
| Marital status (%) |  |  |  |  |  |
| Single/divorced/widowed | 9.3 | 11.2 | 13.1 | 12.5 | 12.0 |
| Married, monogamous | 43.8 | 40.2 | 42.7 | 45.7 | 43.5 |
| Married, polygamous | 46.9 | 48.5 | 44.3 | 41.8 | 44.5 |
| Child sex (%) | 52 | 50.3 | 50.9 | 48.2 | 50.1 |
| Child age at endline (mean (SD) in mo) | 18.0 (0.4) | 18.1 (0.4) | 18.0 (0.4) | 18.0 (0.4) | 18.0 (0.4) |
| Child WLZ at baseline (mean (SD)) | 0.36 (1.21) | 0.27 (1.01) | 0.28 (1.11) | 0.26 (1.08) | 0.29 (1.10) |
| Child LAZ at baseline (mean (SD)) | -1.41 (1.06) | -1.31 (1.07) | -1.39 (0.98) | -1.38 (1.08) | -1.38 (1.04) |

^a^ SQ-LNS=small quantity lipid-based nutrient supplements; WLZ=weight-for-length z-score; LAZ=length-for-age z-score.

**Supplemental Table 4. DYAD-Ghana trial: Summary of baseline characteristics and two**

**concurrent covariates in groups in analysis sample receiving and not receiving LNS**

| **Characteristics** | **No LNS**  **(n=733)** | **20 g LNS**  **(n=360)** | **All**  **(n=1093)** |
| --- | --- | --- | --- |
| Season of interview |  |  |  |
| Rainy | 50.3 | 51.4 | 50.7 |
| Dry | 49.7 | 48.6 | 49.3 |
| Distance to market (%) |  |  |  |
| Nearest quartile | 25.6 | 22.3 | 24.5 |
| Q2 | 24.6 | 27.0 | 25.4 |
| Q3 | 26.7 | 22.8 | 25.4 |
| Farthest quartile | 23.1 | 27.9 | 24.7 |
| Asset index (%) |  |  |  |
| Lowest quartile | 22.8 | 28.1 | 24.5 |
| Q2 | 24.8 | 26.7 | 25.4 |
| Q3 | 27.7 | 25.6 | 27.0 |
| Highest quartile | 24.7 | 19.7 | 23.1 |
| Small livestock score (% with none) | 72.7 | 69.4 | 71.6 |
| HFIA category (%) |  |  |  |
| Food secure | 58.7 | 59.6 | 59.0 |
| Mildly food insecure | 10.1 | 10.9 | 10.4 |
| Moderately food insecure | 18.3 | 17.3 | 18.0 |
| Severely food insecure | 12.9 | 12.3 | 12.7 |
| Number of other underfives |  |  |  |
|  | 57.6 | 53.6 | 56.3 |
|  | 42.4 | 46.4 | 43.7 |
| Maternal education (% with none) | 8.3 | 8.9 | 8.5 |
| Maternal age (mean (SD) in y) | 26.6 (5.4) | 27.0 (5.5) | 26.7 (5.4) |
| Maternal BMI (mean (SD) kg/m2) | 24.7 (4.6) | 25.2 (4.7) | 24.9 (4.7) |
| Main language spoken in household |  |  |  |
| Krobo/Ga | 77.5 | 76.7 | 77.2 |
| Ewe | 11.0 | 13.6 | 11.9 |
| Other | 11.5 | 9.7 | 10.9 |
| Marital status (% formally married) | 40.4 | 36.4 | 39.1 |
| Child sex (% male) | 47.4 | 50.1 | 48.3 |
| Child age at endline (mean (SD) in mo) | 18.1 (0.1) | 18.1 (0.1) | 18.1 (0.1) |

^a^ SQ-LNS=small quantity lipid-based nutrient supplements.

**Supplemental Table 5. DYAD-Malawi trial: Summary of baseline characteristics and two**

**concurrent covariates in groups in analysis sample receiving and not receiving SQ-LNSa**

| **Characteristics** | **No LNS**  **(n=418)** | **20 g LNS**  **(n=207)** | **All**  **(n=625)** |
| --- | --- | --- | --- |
| Season of interview |  |  |  |
| Rainy | 42.1 | 39.7 | 41.3 |
| Harvest | 33.3 | 31.6 | 32.7 |
| Prepare land | 24.6 | 28.7 | 26.0 |
| Distance to market (%) |  |  |  |
| Nearest quartile | 17.3 | 18.2 | 17.6 |
| Q2 | 26.4 | 21.5 | 24.8 |
| Q3 | 30.0 | 31.1 | 30.4 |
| Farthest quartile | 26.4 | 29.2 | 27.3 |
| Asset index (%) |  |  |  |
| Lowest quartile | 29.1 | 22.1 | 26.8 |
| Q2 | 24.8 | 30.3 | 26.6 |
| Q3 | 26.2 | 24.5 | 25.7 |
| Highest quartile | 19.9 | 23.1 | 21.0 |
| Small livestock score (% with none) | 34.7 | 36.7 | 35.4 |
| HFIA category (%) |  |  |  |
| Food secure | 14.5 | 18.3 | 15.8 |
| Mildly food insecure | 11.4 | 13.5 | 12.1 |
| Moderately food insecure | 34.1 | 27.9 | 32.1 |
| Severely food insecure | 40.0 | 40.4 | 40.1 |
| Nulliparous at baseline (%) | 22.6 | 22.6 | 22.6 |
| Maternal education (% with none) | 28.4 | 30.8 | 29.2 |
| Maternal age (mean (SD) in y) | 25.2 (5.9) | 25.4 (6.2) | 25.3 (6.0) |
| Maternal BMI (mean (SD) kg/m2) | 21.5 (2.5) | 21.5 (2.8) | 21.5 (2.6) |
| Maternal HIV status (% HIV+) | 11.8 | 12.0 | 11.9 |
| Ethnicity |  |  |  |
| Yao | 82.0 | 82.3 | 82.1 |
| Chewa | 10.3 | 11.5 | 10.7 |
| Other | 7.7 | 6.2 | 7.2 |
| Marital status (% formally married) |  |  |  |
| Single/divorced/widowed | 9.9 | 11.6 | 10.5 |
| Married, monogamous | 65.4 | 62.8 | 64.5 |
| Married, polygamous | 24.7 | 25.6 | 25.0 |
| Child sex (% male) | 47.6 | 48.3 | 47.9 |
| Child age at endline (mean (SD) in mo) | 18.1 (0.1) | 18.1 (0.1) | 18.1 (0.1) |

^a^ SQ-LNS=small quantity lipid-based nutrient supplements.

**Supplemental Table 6. Covariates significantly associated with outcomes in bivariate models (P<0.10), and included in multivariate modelsa**

| **Outcome** | **ZINC** | **DOSE** | **DYAD-G** | **DYAD-M** |
| --- | --- | --- | --- | --- |
| Still breastfed (not reported fully weaned) | distance to market; maternal age; marital status | distance to market; asset score; number of underfives; maternal education; ethnicity, baseline infant LAZ | assets; small livestock score; maternal education, maternal age | season; distance to market; asset score; small livestock score; household food insecurity; maternal HIV; maternal education; ethnicity |
| Breastfed 6 or more times yesterday | season; distance to market; maternal education; maternal age; maternal BMI; ethnicity | distance to market; asset score; number of underfives; maternal education; maternal age | maternal education, maternal age | season; distance to market; asset score; household food insecurity; maternal education; ethnicity |
| Met WHO minimum for number of feeding episodes yesterday | asset score, maternal education; ethnicity; infant age | *No data for outcome* | *No data for outcome* | Small livestock score |
| 4 or more food groups yesterday | season; asset score; small livestock score; ethnicity; infant sex; infant age | distance to market; asset score; small livestock score; household food insecurity; maternal education; male; baseline infant WLZ | distance to market, maternal education, main language, infant sex | distance to market; asset score; small livestock score; household food insecurity, maternal education; maternal BMI; ethnicity; infant age |
| Number of animal-source food groups yesterday, range 0-5 | season; asset score; small livestock score, household food insecurity; marital status; ethnicity | distance to market; asset score; household food insecurity; maternal education; maternal BMI; ethnicity; marital status; infant age | distance to market, asset score; maternal education, maternal BMI, main language, marital status | season; distance to market; asset score; small livestock score; household food insecurity; maternal education; maternal age; maternal BMI; ethnicity |
| Number of fruit/vegetable food groups yesterday, range 0-5 | season; asset score; maternal education; maternal BMI; ethnicity; infant age | season; distance to market; household food insecurity; baseline infant WLZ | asset score; main language | distance to market; ethnicity |
| Animal-source food score last 7 d, range 0-28 | season; asset score; small livestock score; household food insecurity; number of underfives; maternal education; maternal BMI; ethnicity | season; distance to market; asset score; household food insecurity; maternal education; ethnicity; marital status; infant age; baseline infant LAZ | asset score; maternal education, maternal age, maternal BMI, main language, marital status, infant age | distance to market; asset score; small livestock score; household food insecurity; maternal HIV; maternal education; maternal age; maternal BMI; ethnicity; marital |
| Fruit/vegetable score last 7 d, range 0-35 | season; asset score; number of underfives; maternal education; maternal BMI; ethnicity; infant age; baseline infant LAZ | distance to market; asset score; household food insecurity; maternal education; ethnicity | asset score; food insecurity, maternal education, maternal age, main language, marital status | season; distance to market; asset score; household food insecurity; maternal education; ethnicity |
| Lowest tertile for 7 d ASF score | season; distance to market; asset score; household food insecurity; maternal education; ethnicity | season; distance to market; asset score; household food insecurity; maternal education; infant age | asset score; maternal education, maternal age, maternal BMI, main language, marital status | distance to market; asset score; parity; maternal education; maternal age; infant age |
| Lowest tertile for 7 d fruit/vegetable score | season; asset score; maternal education; ethnicity; infant age | asset score; household food insecurity; number of underfives; maternal education | season, distance to market, asset score; food insecurity, maternal education, main language, marital status | season; distance to market; asset score; household food insecurity; maternal education; ethnicity |

^a^ ZINC is the iLiNS-ZINC trial in Burkina Faso; DOSE is the iLiNS DOSE trial in Malawi; DYAD-G is the iLiNS DYAD trial in Ghana; DYAD-M is the iLiNS DYAD trial in Malawi.

**Supplemental Figures 1-4**

**LNS – 25 communities^a^**

**2435 infants**

**No LNS – 9 communities**

**785 infants**

**3402 screened**

**Exclusions 182**

55 no parental consent

55 excluded due to unavailability

26 excluded due to physical condition

46 excluded one of two twins

**3220 infants enrolled**

**18 mo postpartum**

1957 infants in analysis sample

**18 mo postpartum**

665 infants in analysis sample

**475 infants dropped out**

33 died

442 lost to follow-up

**3 infants missing data**

**119 infants dropped out**

25 died

94 lost to follow-up

**1 infant missing data**

^a^ The ZINC study design for primary outcomes entailed randomization of communities where children received LNS to 4 levels of zinc in 20 g LNS and a zinc tablet or placebo. See Hess et al. 2015 for details. For purposes of this analysis, all 4 groups receiving 20 g LNS were combined and compared to the group not receiving LNS.

**Supplemental Figure 1.** iLiNS Burkina ZINC study flow diagram

**No LNS – 320 infants**

**2136 screened**

**Exclusions 182**

53 older than 6.5 mo at screening

110 younger than 5.5. mo

7 out of catchment area

20 refused

14 not known

**1932 infants enrolled**

**18 mo postpartum**

227 infants in analysis sample

**62 infants dropped out**

15 died

47 lost to follow-up

**31 infants missing data**

**10 g LNS – 321 infants**

**20 g LNS – 645 infants^a^**

**40 g LNS – 646 infants^a^**

**129 infants dropped out**

- 18 died
- 111 lost to follow-up

**73 infants missing data**

**80 infants dropped out**

- 13 died
- 67 lost to follow-up

**42 infants missing data**

**129 infants dropped out**

- 18 died
- 111 lost to follow-up

**73 infants missing data**

**18 mo postpartum**

- 199 infants in analysis sample

**18 mo postpartum**

- 443 infants in analysis sample

**18 mo postpartum**

- 449 infants in analysis sample

^a^ The DOSE study design for primary outcomes entailed randomization into six groups, with the 20 g and 40 g LNS groups each divided into a group receiving LNS with milk and a group receiving non-milk containing LNS. See Maleta et al. 2015 for details. For purposes of this analysis, the two groups receiving 20 g LNS were combined and the two groups receiving 40 g LNS were combined.

**Supplemental Figure 2.** iLiNS Malawi DOSE study flow diagram

**MMN - 439 women**

**IFA - 441 women**

**LNS - 440 women**

**351 not recruited**

108 planned to move

95 refused

55 not found

45 lived outside study area

20 >20 weeks gestation

13 husband refused

9 loss of pregnancy

4 unwilling to take supplement

2 sickness

**2607 women screened**

**681 not eligible**

224 lived outside study area

203 HIV positive

96 >20 weeks gestation

67 asthma

56 planning move

9 unwilling to receive visitors

7 misdiagnosed pregnancy

4 participating in a clinical trial

4 less than 18 years

3 chronic respiratory disease

2 suspected peanut allergy

2 epilepsy

2 loss of pregnancy

1 unwilling to take study supplement

1 redundant (enrolment ended)

**1926 women eligible**

**1575 women recruited**

**1320 women enrolled^a^**

**255 not enrolled**

120 > 20 weeks gestation after ultrasound

77 >20 weeks before completion of enrolment

12 husband refused

11 woman refused

9 loss of pregnancy

9 misdiagnosed pregnancy

8 acute illness

5 redundant (enrolment ended)

1 in-law refused

1 moved away

1 planning to move away

1 mental illness

**408 infants enrolled**

**411 infants enrolled**

**409 infants enrolled**

**18 mo postpartum**

372 infants in analysis sample

**18 mo postpartum**

361 infants in analysis sample

**18 mo postpartum**

360 infants in analysis sample

**33 infants not enrolled**

15 miscarriage /abortion

12 stillbirth

3 mother moved

2 mother refused

1 misdiagnosed pregnancy

**15 infants dropped out**

7 died

4 parent moved

4 parent refused

**21 infants missing data**

**10 infants dropped out**

8 died

1 parent moved

1 parent refused

**40 infants missing data**

**18 infants dropped out**

12 died

2 parent moved

4 parent refused

**31 infants missing data**

**28 infants not enrolled**

10 miscarriage /abortion

10 stillbirth

6 mother moved

2 mother refused

**31 infants not enrolled**

12 miscarriage /abortion

7 stillbirth

7 mother moved

1 mother refused

4 misdiagnosed pregnancy

^a^ The DYAD study design for primary outcomes entailed randomization into 3 groups as shown. For the present analysis, we planned *a priori* to compare those that did not receive infant SQ-LNS (i.e. the IFA and MMN groups, combined) to the SQ-LNS group.

**Supplemental Figure 3.** iLiNS DYAD-Ghana study flow diagram

**MMN – 291 women**

**IFA – 290 women**

**LNS – 288 women**

**9310 approached**

**Exclusions 7919**

3470 not interested

2760 out of area

1333 >20 gest weeks or duration unknown

310 not available

9 underage

1 earlier participation

30 medical condition

6 other

**1391 women enrolled**

**522 assigned to pregnancy only intervention**

**869 for full follow-up^a^**

**265 infants enrolled**

**266 infants enrolled**

**266 infants enrolled**

**18 mo postpartum**

208 infants in analysis sample

**18 mo postpartum**

210 infants in analysis sample

**18 mo postpartum**

207 infants in analysis sample

**29 infants not enrolled**

1 miscarriage /abortion

8 stillbirth

20 lost to follow-up

**36 infants dropped out**

18 died

18 lost to follow-up

**21 infants missing data**

0 twins excluded

**33 infants dropped out**

15 died

18 lost to follow-up

**23 infants missing data**

0 twins excluded

**30 infants dropped out**

9 died

23 lost to follow-up

**25 infants missing data**

2 twins excluded

**27 infants not enrolled**

2 miscarriage /abortion

1 stillbirth

24 lost to follow-up

**36 infants not enrolled**

4 miscarriage /abortion

8 stillbirth

24 lost to follow-up

^a^ The DYAD study design for primary outcomes entailed randomization into 3 groups as shown. For the present analysis, we planned *a priori* to compare those that did not receive infant SQ-LNS (i.e. the IFA and MMN groups, combined) to the SQ-LNS group.

**Supplemental Figure 4.** iLiNS DYAD-Malawi study flow diagram
